# The optimal montage to mark interictal epileptiform discharges and high-frequency oscillations in intraoperative electrocorticography

**DOI:** 10.1016/j.cnp.2025.06.007

**Published:** 2025-07-01

**Authors:** Ziyi Wang, Jiaojiao Guo, Eline Schaft, Sem Hoogteijling, Cyrille H. Ferrier, Gerhard H. Visser, Dongqing Sun, Friso Hoefnagels, Taku Inada, Sandra van der Salm, Geertjan Huiskamp, Nicole van Klink, Maryse van’t Klooster, Maeike Zijlmans, On behalf of the RESPect database study group

**Affiliations:** a Department of Neurology and Neurosurgery, University Medical Center Utrecht Brain Center, University Medical Center Utrecht, Part of ERN EpiCARE, P.O. box 85500, 3508 GA Utrecht, the Netherlands; b Stichting Epilepsie Instellingen Nederland (SEIN), the Netherlands

**Keywords:** Epilepsy surgery, Intraoperative electrocorticography, Montage, Electrode grid, High-frequency oscillations

## Abstract

**Objective:**

In intraoperative electrocorticography (ioECoG), interictal epileptiform discharges (IEDs) and high-frequency oscillations (HFOs; ripples 80–250 Hz, fast ripples (FRs) 250–500 Hz) can be identified in *average* or *bipolar* montage. We studied how montage choice affects event identification.

**Methods:**

Two reviewers independently marked IEDs and HFOs across three montages (*average,* horizontal- and vertical-*bipolar*) from 13 patients who were seizure-free after ioECoG-guided surgery. We analyzed the number of channels-with-events, total events count, events morphology (maximum-amplitude, duration, frequency), number of instances with overlapping events across multiple channels (event_instance), concordance of event_instances over montages, and percentage of channels-with-events in the resected-area.

**Results:**

*Bipolar* montages yielded more channels-with-events, higher counts, and greater maximum-amplitude of IEDs and ripples compared to *average* montages. *Average* and horizontal*-bipolar* montages yielded more IED_instances than vertical*-bipolar* montages. *Average* montages detected the highest percentage of event_instances occurring only in this montage. Event duration, frequency, and percentage of channels-with-events in the resected-area did not differ across montages.

**Conclusions:**

All three ioECoG montages are clinically useful to find epileptic events. The *bipolar* montage detects more events with greater amplitude, while the *average* montage uncovers a wider variety of unique events. Combining montages provides complementary information.

**Significance:**

This study quantitatively revealed how different montages capture epileptiform events.

## Highlights

• *Bipolar* montage yields more and higher-amplitude interictal epileptiform discharges and ripples than *average* montage.
• *Average* montage captures more unique interictal epileptiform discharges and high-frequency oscillations than *bipolar* montage.
• No clinically relevant difference was found in the number of channels-with-events in the resected-areas.

## 1 Introduction

Epilepsy surgery offers a chance for cure for people with refractory epilepsy (Fois et al., 2016, Zijlmans et al., 2019). Surgical effectiveness hinges on precise delineation and complete resection of epileptogenic tissue (Zijlmans et al., 2019). Intraoperative electrocorticography (ioECoG) directly measures activity from the cortex and enables repeated recordings, enhancing surgical precision by identifying electrophysiological abnormalities (Guo et al., 2024, Zweiphenning et al., 2022).

Conventional interictal epileptiform discharges (IEDs) are well-established biomarkers of epilepsy (Alarcon et al., 1997). IEDs are defined as transient events characterized by a 'spike' or sharp waveform lasting 20–70 ms, distinctively standing out from the background brain activity (Kural et al., 2020a, Kural et al., 2020b). High-frequency oscillations (HFOs), subdivided into ripples (80–250  Hz) and fast ripples (FRs; 250–500  Hz), have been proposed as a novel biomarker to more precisely localize the source of the epilepsy than IEDs (van't Klooster et al., 2015, van't Klooster et al., 2017, van Klink et al., 2014, van Klink et al., 2021, Wang et al., 2024, Zijlmans et al., 2012b). HFOs are defined by their morphology, defined as at least four consecutive oscillations with an amplitude clearly above the baseline signal, and a duration of less than 200 ms (Burnos et al., 2016, Mari et al., 2012, Zelmann et al., 2012).

Various EEG display montages are used to capture seizures and epileptiform events in clinical EEG practice (Duce et al., 2014, Foldvary et al., 2000). The most prevalent montages are: a *common* (external) *referential*, a standard *average*, and a *bipolar* montage (Acharya and Acharya, 2019). Similar to surface EEG, a *common referential* montage is used for both recording and clinical review purpose during ioECoG monitoring (Pickard and Skidmore, 2019). It involves a reference electrode that is often placed on the contralateral mastoid. This hardware setup, with the considerable distance between the reference point and the electrodes on the grid, is susceptible for noise or artifacts. This issue can be addressed by transforming the raw data into standard *average* or *bipolar* montage for a clearer display (Boran et al., 2019, Ferrari-Marinho et al., 2015, Weiss et al., 2018).

**Fig. 1:**
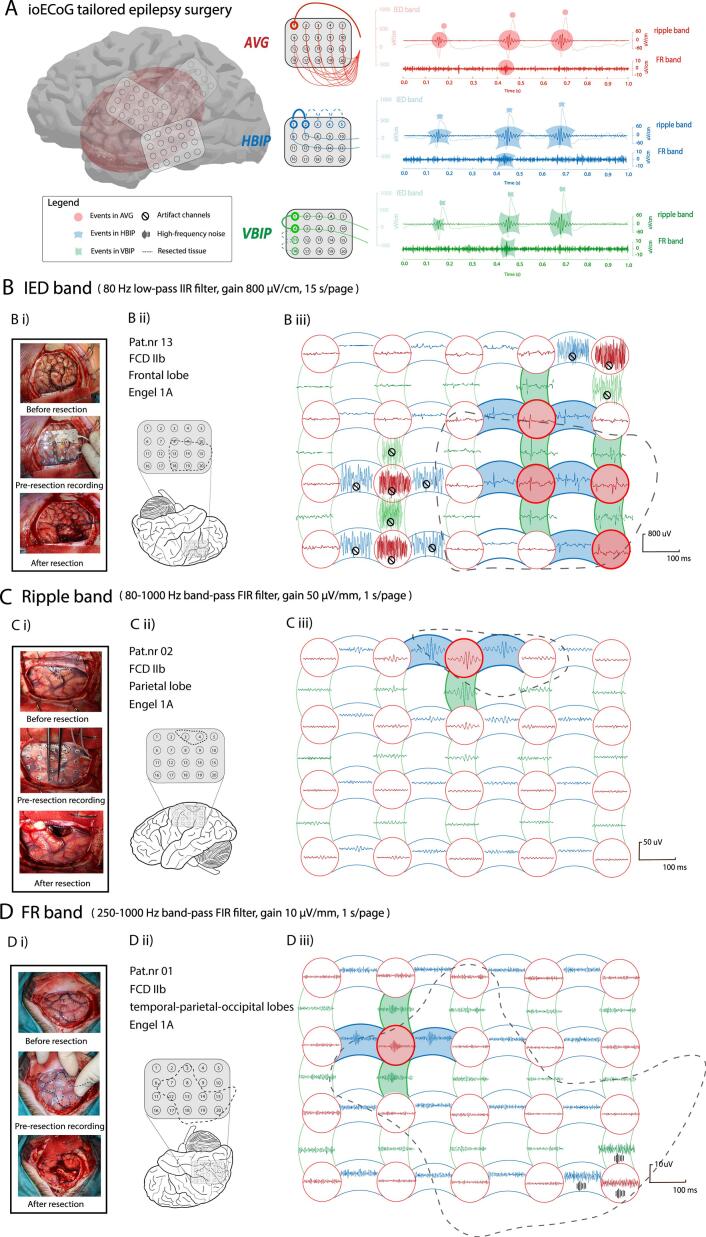
**Schematic illustration of the ioECoG recording situations, the three montages with their corresponding channels, and three representative patient examples. A. Schematic representation of a standard electrode grid configured with *AVG*, *HBIP*, and *VBIP* montages.** After opening the dura, the grid is placed in various positions per stage, and ioECoG is recorded in each position for 2–5 min. Based on the standard 4x5 grid configuration, the AVG consist of 20 channels, the HBIP of 16 channels, and the VBIP of 15 channels. An example of data from a single channel, showing events in the IED, ripple, and fast ripple (FR) band, is presented for each montage. **AVG (in red):** average reference signal is calculated by summing the voltages from all electrodes (excluding the mastoid reference, and bad channels) and dividing this by the total number of electrodes. The potential for each electrode is calculated by subtracting the average reference signal from the voltage recorded at that electrode. **HBIP (in blue):** for each neighboring pairs of adjacent electrodes that are transversely aligned − left to right in horizontal direction over the grid −, the bipolar potential is calculated as the voltage recorded at the left electrode subtracted from the voltage recorded at the right electrode (e.g., 01-02, 02-03, 03-04, 04-05). **VBIP (in green):** for each neighboring pair of adjacent electrodes that are longitudinally aligned − top to bottom in vertical direction over the grid −, the bi-polar potential is calculated as the voltage recorded at the lower electrode subtracted from the voltage recorded at the upper electrode (e.g., 01-06; 06-11; 11-16). **B. Patient example (Pat.nr 13) showing IEDs in the frontal lobe for the three montages. Bi)** intra-operative photographs (before-resection, pre-resection recording, and after-resection) are matched to determine channel positions and resected tissue (also for Ci) and Di)). **Bii)** patient characteristics (Pat.nr, histopathology, anatomical location, 1y seizure outcome) and schematic visualization of the pre-resection grid position including the resected tissue area to label channel as either resected or non-resected (also for C ii) and D ii)). **Biii)** in the pre-recording, IEDs were observed on *AVG* channel 09, 14, 15 and 20; on *HBIP* channel 08–09, 09–10, 13–14, 14–15, and 19–20; on *VBIP* channel 04–09, 09–14, 14–19, 10–15, 15–20. Instantaneous artifacts were observed on *AVG* channel 05, 12, 17; on *HBIP* channel 04–05, 11–12, 12–13, 16–17, 17–18; on *VBIP* channel 05–10, 07–12, 12–17. **C**. **Patient example (Pat.nr 02) showing ripples in the parietal lobe for the three montages. Ciii)** in the pre-recording, ripples were observed on *AVG* channel 03; on *HBIP* channel 02–03, 03–4; on *VBIP* channel 03–08. **D. Patient example (Pat.nr 01) showing FRs in the temporal-parietal-occipital region for the three montages. Diii)** in the pre-recording, FRs were observed on *AVG* channel 07; on *HBIP* channel 06–07, and 07–08; on *VBIP* channel 02–07, 07–12. Instantaneous high-frequency noise were observed on *AVG* channel 20; on *HBIP* channel 19–20; on *VBIP* channel 15–20. *Abbreviations*: *AVG* = *average* montage; *HBIP* = horizontally-oriented *bipolar* montage; *VBIP* = vertically-oriented *bipolar* montage; IED = interictal epileptiform discharge; FR = fast ripple; ioECoG = intraoperative electrocorticography; Pat.nr = patient number; FCD = focal cortical dysplasia; Engel 1A = seizure free ≥ 1 year after surgery. (For interpretation of the references to colour in this figure legend, the reader is referred to the web version of this article.)

An *average* montage generally preserves the broader shape and amplitude distribution of IEDs across its coverage, allowing for better visibility of electrical activity (Acharya and Acharya, 2019). Yet on the down-side, the averaging process can smooth out the sharp features of IED waveforms. Further, artifacts in one channel can contaminate all other channels when using an uncorrected *average* montage (Acharya and Acharya, 2019, Acharya et al., 2016). Stereo-EEG is often reviewed in a *bipolar* montage and it enables direct channel-wise localization of epileptic activity (Caune et al., 2013, Michelmann et al., 2018). In line with the pioneering stereo-EEG studies (Bagshaw et al., 2009, Melani et al., 2013), we and other centers used *bipolar* montages for the detection of HFOs on ioECoG (Boran et al., 2019, Weiss et al., 2021, Zweiphenning et al., 2022). In an ioECoG grid configuration, both horizontal and vertical directions along the grid are possible (Fig. 1A). The *bipolar* configuration depends on potential differences between two adjacent recording electrodes (Acharya et al., 2016) and has the advantage of eliminating common noise, but might also mask identical events occurring on two neighboring electrodes (Pickard and Skidmore, 2019).

Marking intraoperative events can be done in any aforementioned montage configuration, but it is an unanswered question whether different montages capture equivalent or akin IEDs and HFOs. This work compared IEDs and HFOs marked in ioECoG recordings using three types of montage configurations: *average* montage *(AVG)*, horizontally-oriented *bipolar* montage *(HBIP)*, and vertically-oriented *bipolar* montage *(VBIP)*). Our main research purpose was to pinpoint the methodological issues related to diverse ioECoG montages, and how these influence the accurate identification and interpretation of IEDs and HFOs.

## 2 Methods

### 2.1 Study design and patient selection

We retrospectively selected 13 people with refractory focal epilepsy characterized by a neocortical lesion who were part of the Registry for Epilepsy Surgery Patients (RESPect) database of the University Medical Center Utrecht (UMC Utrecht) and Stichting Epilepsie Instellingen Nederland (SEIN). Those 13 people underwent ioECoG tailored epilepsy surgery between 2008 and 2018, and achieved seizure freedom (Engel 1A) at ≥ 1y. We assumed that seizure-freedom signified complete resection of the presumed epileptogenic tissue. Our aim was to assess various aspects of the aforementioned montages in a diverse population, ensuring representation across: 1) all anatomical regions of the brain (i.e. temporal, frontal, parietal, and occipital), excluding patients with mesial temporal lobe epilepsy due to the inability to visualize grid placement; 2) common underlying histopathologies associated with epilepsy (e.g. malformations of cortical development (MCD) and central nervous system (CNS) tumors.

The RESPect database was approved by the institutional ethical committee of UMC Utrecht, who exempted the requirement for written informed consent till 2018 due to the research’s retrospective nature, whereas explicit informed consent is requested from 2018 onwards (non-WMO; METC18-109C). All collected data were coded to maintain (pseudo)anonymity.

### 2.2 Surgical procedure and ioECoG recordings

Epilepsy surgery was performed under general intravenous propofol anesthesia. The neurosurgeon exposed the cortex intended to be removed and a few centimeters of the surrounding tissue. We recorded ioECoG with standard 4x5 grid electrodes (Ad-Tech, Racine, WI), featuring platinum surfaces encased in silicone with a contact area of 4.2 mm^2^ and an inter-electrode distance of 10 mm. The ioECoG was recorded using a 32 or 64-channel EEG system (Micromed, Treviso, Italy) at 2048 Hz sampling rate, with an 538 Hz antialiasing filter. Recorded signals were referenced to an external electrode positioned on the contralateral mastoid. To reduce the potential interference of burst suppression, we temporarily halted the administration of propofol before ioECoG monitoring (Sun et al., 2024).

IoECoG recordings composed three stages: 1) Pre-resection recordings: grids were positioned in multiple locations before the planned resection to encompass all suspected epileptogenic tissue; 2) Intermediate-resection recordings: if epileptic activity persisted after the initial resection, the resection was extended if not limited by eloquent cortex or too distant locations; 3) Post-resection recordings: following the finalized resection, ioECoG was recorded from cortex around the cavity. The ioECoG was recorded in each position for 2–5 min, consistent with our previous work (Schaft et al., 2024, Zweiphenning et al., 2022, van Klink et al., 2021). Intraoperative photographs and crafted sketches were used to document the ioECoG position on the cortex. IoECoG electrode placement was guided by surgical practicalities and was not standardized across patients. For example, the neurosurgeons tend to place electrode grids in a way that maximizes contact with regions suspected of epileptogenic activity, while fitting within the boundaries of the craniotomy, thus not typically oriented parallel or perpendicular to the gyri configurations. In temporal lobe epilepsy, the grid was often placed first directed towards the temporal pole and then towards dorsotemporal. In those situations, the horizontal configuration generally follows the natural pattern of the temporal gyri. IoECoG guided surgical resection of the epileptogenic tissue was based on preoperative MRI findings and IEDs. One patient was enrolled in a RCT “the HFO trial” (Zweiphenning et al., 2022) and assigned to the HFO-guided tailoring arm, meaning that HFOs instead of IEDs were used to guide the surgeon.

### 2.3 Montage configurations, artefacts and channel labels

We re-referenced the raw data offline to *average* (*AVG)*, horizontally-oriented *bipolar* (*HBIP),* and vertically-oriented *bipolar (VBIP)* montages (Fig. 1A). Channels in each montage that exhibited continuous artefact interference or no cortical signal were excluded from further analysis.

We used custom-made software to align pre- and intermediate-resection photographs to post-resection photographs, identifying which unipolar grid electrodes in pre- and intermediate- resection recordings covered the eventually resected-area (Fig. 1Bii, 1Cii, and 1Dii). We categorized unipolar channels into two groups: i) *Resected*, covering the complete post-resection cavity, which encompassed tissue ≤ 5 mm inward from the edge of resection cavity; ii) *Non-resected*, covering the tissue > 5 mm from the edge of the resection cavity. Based on these unipolar labels, we derived bipolar labels: i) *Resected*, if at least of one of the two unipolar channels were resected; ii) *Non-resected*, if both unipolar channels were non-resected. In both intermediate- and post-resection recordings, we excluded all unipolar channels located above the resection cavity and their corresponding bipolar channels.

### 2.4 IEDs and HFOs markings

We selected one-minute epochs at the end of each recording to minimize propofol effects. (Sun et al., 2024, Zijlmans et al., 2012a) All events were marked in BrainQuick Software (Micromed, Treviso, Italy) in three montages (i.e. *AVG*, *HBIP,* and *VBIP*)*.* The reviewers were restricted to using only one montage during the marking process. IEDs were marked independently, blinded for HFOs. IEDs were marked using the consistent settings across three montages: 80 Hz low-pass IIR filter, gain 800 µV/cm, 15 s/page. We defined IEDs as transient and stereotype waveforms (< 80 Hz) distinctly standing out from the baseline with an evident epileptiform pattern, including single spikes (< 80 ms), shape waves (> 80 ms), periodic spikes/ sharp waves, poly-spikes, and spike/sharp wave and-slow wave complexes. IEDs were marked separately by two reviewers (ZW, JG) and checked by two experts (MvtK, CF).

HFOs were initially marked in the three montages by an automated HFO detection algorithm, (Boran et al., 2019, Fedele et al., 2016). Consistent settings across montages were used: ripple: 80–1000 Hz band-pass FIR filter, gain 50 µV/mm, 1 s/page; FR: 250–1000 Hz band-pass FIR filter, gain 10 µV/mm, 1 s/page). All automated marked events were visually inspected separately by two reviewers (ZW, JG) on two 22-inch wide screens. One screen displaying ripples and the other showing FRs, both time-locked for simultaneous viewing. Three HFO experts (MvtK, MZ, NvK) checked the marked HFOs.

### 2.5 Interrater agreement

The interrater agreement between the two reviewers (ZW, JG) was calculated using Cohen’s Kappa coefficient (κ-value) for the visually marked IEDs and HFOs per 1-minute segment across all three montages (Dewey, 1983). A κ < 0 reflects that the agreement was made by chance; κ = 0.5 reflects moderate agreement; κ = 1 indicates complete agreement. We defined κ > 0.5 as the threshold of sufficient concordance. (Landis and Koch, 1977).

Agreed-events (IEDs, ripples, and FRs) were defined as events marked by both reviewers (ZW and JG). Recordings with more agreed-events had a greater influence on the calculation of the κ-value for that montage compared to recordings with fewer agreed-events. To address this imbalance, we calculated the overall weighted average κ-value as a proxy for IED and HFO marking quality across the three montages. For example,

$The\;overall\;weighted\;average\;\kappa -value\;for\;the\;IED\;in\;AVG\;=\frac{\left(\kappa value\_1*Nr.AgreedIED\_1\right)+\left(\kappa value\_2*Nr.AgreedIED\_2\right)+\left(\kappa value\_3*Nr.AgreedIED\_3\right)+\cdots }{\mathrm{Nr}.AgreeIED1+Nr.AgreeIED2+Nr.AgreeIED3+\cdots }$

1,2,3⋯represented the recording numbers. All agreed-events, onwards termed ‘events’, were retained for subsequent montage performance analysis, while events marked by only one reviewer (ZW or JG) were rejected.

### 2.6 Montage performance and analysis

All events were extracted from the BrainQuick.trc files and imported into Matlab (version 2022, The MathWorks Inc, Natick, MA) for further analysis. We assessed the montage performance as follows: 1.) number of channels-with-events; 2.) total event counts; 3) number of event_instances; 4.) concordance of event_instance over montages; 5.) events morphology; 6.) percentage of channels-with-events located within the resected-area. The assessments were performed independently for IEDs, ripples, and FRs, and subsequently compared across the three montages.

1) Number of channels-with-events: we normalized for the variance in the total number of artefact-free channels across the montages by using the number of artefact-free channels in *AVG* montage as reference. Based on this reference, we proportionally adjusted the channel counts in the *HBIP* and *VBIP* montages. We first calculated the normalized number of channels-with-events for each recording (pre-resection, intermediate, and post-resection) at the recording group-level. We further summed these number across all recordings for each patient, obtaining patient group-level data.
2) Total events count: following the same proportional adjustment based on channel normalization, we calculated the normalized event counts at the recording- and patient- group levels.
3) Number of event_instances: we calculated the number of “event_instances” during which events overlapped across multiple channels at recording group-level.
4) Concordance of event_instances over montages: for each event_instance identified in 3) at the recording group-level, we determined in which montage this event_instance was detected. Simultaneous event_instances in at least two montages were classified as “concordant event_instances”. In contrast, event_instances that were detected solely in one montage were classified as “unique event_instances”. Venn plots were used to illustrate the concordant and the unique event_instances between the montages.
5) Event morphology: we calculated for each event; i) the maximum-amplitude (in µV), identifying the highest absolute value within the duration of the event based on Hilbert transform envelope; ii) the duration (in ms), from start to stop of the marking of the event; iii) the frequency (in Hz), counting the number of times an event crosses the zero horizontal axis (zero crossing rate). For further comparisons, we used the averaged morphologies, weighted at the recording group-level. The event count for each recording represented its relative importance, with recordings containing a higher event count carrying greater statistical weight.
6) Percentage of channels-with-events located within the resected-area: we calculated the amount of channels-with-events located inside the resection margin, for each pre- and intermediate-recording. We then compared the percentage as following:

$The\;percentage\;channels\;with\;event\;in\;resected\;area\;=\frac{\mathrm{The}\;amount\;of\;channels\;with\;events\;in\;the\;resected\;area}{\mathrm{Total}\;amount\;of\;channels\;in\;the\;resected\;area}$

### 2.7 Statistical analysis

We calculated and compared the following variables among three montages: i) weighted average κ-values; ii) number of channels-with-events; iii) total events count; iv) number of event_instances; v) event morphology (maximum-amplitude/duration/frequency); vi) percentage of channels-with-events in the resected-area.

These variables contained three-level nested data, namely *several recordings* (*i*) within *multiple recording stages* (e.g pre-, intermediate-, and post- recordings (*R*)) *per patient* (*P*). Two generalized linear mixed models (GLMM) were utilized to account for these dependencies:

- Model 1 used for the analysis of i) and v):

log(E[Y*_m__i__RP_*]) = (β0 + γ0 + δ0) + (β1X*_Hm_* + β2X*_Vm_* + β3X*_i__RP_*)+ (∊*_mRP_* + ζ_RP_ + η_P_)

- And Model 2 used for the analysis of ii), iii), iv), and vi):

log(E[Y*_miRP_*]) = (β0 + γ0 + δ0) + (β1X*_Hm_* + β2X*_Vm_*)+ (∊*_mRP_* + ζ_RP_ + η_P_)

where (**log(E[Y*_miRP_*])** is the logarithmic transformation to the calculated variable for *recording i* in *recording-stage R* in *patient P* on *montage m*, **β0** the overall intercept; **γ0** the fixed effect for *recording-stage R*; ***δ*0** the fixed effect for *patient P*; **X*_Hm_*** & **X*_Vm_*** dummy variables for *HBIP* and *VBIP* montage (*AVG* was selected as the reference category); **X*_i RP_*** (weighted power) the count of events for *recording i* in *recording-stage R* in *patient P*; **∊*_mRP_*** the residual error *for recording i* in *recording-stage R* in *patient P* on *montage m*; **ζ_RP_** the residual error for *recording-stage R* in *patient P*; **η_P_** the residual error for *patient P* **β1, β2,** and **β3** represent the variable coefficients.

We reported continuous variables as medians with interquartile ranges (IQR). Continuous variables were assessed using either the Chi-square test or Fisher's exact test with Bonferroni correction. Due to the non-normal distribution of the event counts at the patient group-level, we employed non-parametric Friedman tests to examine differences across the three aforementioned montages. Post-hoc Nemenyi test was conducted following the Friedman test for pairwise comparisons. In the context of GLMM, we used Estimate value (ES) to represent the log-transformed difference between groups, with exponentiated values (i.e., exp(Estimate) indicating multiplicative changes in the expected outcome, where positive values indicate an increase and negative values indicate a decrease. Tukey adjustment was applied following the GLMM model to account for multiple comparisons and control family-wise error rates. All statistical analyses were performed using RStudio software 4.1.0 version. Results were considered significant for p-values < 0.05.

## 3 Results

### 3.1 Patient population and ioECoG recordings

We enrolled 13 patients (6 females, median age at surgery 10 years [range:1- 53y]; Table 1) of whom four had an epileptic focus located in the frontal lobe, two in the temporal lobe, and seven at the intersection of these regions. Nine patients underwent a single stage resection, four underwent multiple stages resection with intermediate-recordings. The two most common underlying pathologies were MCDs and CNS tumors. A total of seventy-nine recordings (40 pre-, 10 intermediate-, and 29 post-resection recordings) from 13 patients were analyzed. Across these recordings, 1373 artifact-free channels in the *AVG* montage, 1041 in the *HBIP* montage, and 967 in the *VBIP* montage were included. (Table 2) Three representative ioECoG examples displaying each of the three epileptiform events (IEDs, ripples, and FRs) were chosen from three individual patients to illustrate consistencies and discrepancies among montages (Fig. 1Biii, 1Ciii, and 1Diii).

**Table 1:** Patient population.

**Pat.nr**	**Sex**	**Age at surgery**	**Anatomical Location (side)**	**Histopathology** **(type)**	**No. of Recordings per stage**			**Post-surgical follow-up** **(years)**
					**Pre**	**Intermediate**	**Post**	
01	F	9	Temporo-parieto-occipital (R)	MCD (FCD IIb)	4	4	1	SF (1)
02	F	17	Frontal (L)	MCD (FCD IIb)	1	NA	1	SF (2)
03	M	3	Frontal (R)	MCD (FCD IIb)	4	NA	3	SF (1)
04	M	7	Temporal-parietal (R)	CNS tumor (Neuronal and mixed-neuronal-glial tumor)	1	1	1	SF (3)
05	M	10	Temporal-parietal (L)	CNS tumor (Neuronal and mixed-neuronal-glial tumor)	5	NA	4	SF (4)
06	M	14	Frontal (L)	MCD (FCD IIb)	4	NA	4	SF (2)
07	F	13	Temporal (L)	CNS tumor (Neuronal and mixed-neuronal-glial tumor)	3	2	1	SF (2)
08	F	10	Fronto-parietal (R)	Others	1	NA	1	SF (1)
09	M	5	Pariel-occipital (L)	MCD (FCD IIb)	6	NA	3	SF (7)
10	F	16	Frontal (L)	MCD (FCD IIa)	2	NA	1	SF (1)
11	F	53	Fronto-parietal (R)	Others	2	NA	1	SF (7)
12	M	10	Temporal (R)	CNS tumor (Neuronal and mixed-neuronal-glial tumor)	3	NA	3	SF (2)
13	M	1	Fronto-parietal (L)	MCD (FCD IIb)	4	3	5	SF (2)

Abbreviations: Pat.nr = the number of patient; R = right side; L = left side; MCD = malformations of cortical development; FCD = focal cortical dysplasia; CNS = central nervous system; NA = not available; SF = seizure free; AVG = standard average montage; HBIP = horizontally-oriented bipolar montage; VBIP = vertically-oriented bipolar montage.

**Table 2:** Agreed-events marked by both reviewers.

	**AVG**	**HBIP**	**VBIP**	**P-value**^#^
**IEDs***(marked in)*				
**No. of patients / Total No. of patients (%)**	11/13 (84.6 %)	10/13 (77 %)	11/13 (84.6 %)	1.00
**No. of recordings / Total No. of recordings (%)**	50/79 (63.2 %)	50/79 (63.2 %)	53/79 (67.1 %)	1.00
**No. of channels / Total No. of channels (%)**	441/1373 (32.1 %)	341/1041 (33.2 %)	302/967 (31.2 %)	0.76
**Total No. of aIEDs**	9555	9003	7624	N/A
				
**Ripples***(marked in)*				
**No. of patients / Total No. of patients** **(%)**	13/13 (100 %)	13/13 (100 %)	13/13 (100 %)	N/A
**No. of recordings / Total No. of recordings (%)**	72/79 (91.1 %)	72/79 (91.1 %)	72/79 (91.1 %)	1.00
**No. of channels / Total No. of channels (%)**	405^a^/1373 (29.4 %)	431^b^/1041 (41.4 %)	409^b^/967 (42.3 %)	<0.0001*
**Total No. of ripples**	1791	2098	1814	N/A
				
**FRs***(marked in)*				
**No. of patients / Total No. of patients** **(%)**	5/13 (38.4 %)	4/13 (30.7 %)	5/13 (38.4 %)	1.0
**No. of recordings / Total No. of recordings (%)**	14/79 (17.7 %)	9/79 (11.4 %)	11/79 (11.4 %)	0.52
**No. of channels / Total No. of channels (%)**	21/1373 (1.53 %)	25/1041 (2.40 %)	23/ 967 (2.48 %)	0.36
**Total No. of FRs**	42	44	45	N/A

Abbreviations: AVG = *standard average* montage; HBIP = horizontally-oriented bipolar montage; VBIP = vertically-oriented bipolar montage; No = Number; N/A = Not applicable; IED: interictal epileptiform discharge; FR: fast ripple.

^#^ Pairwise comparisons were conducted using the chi-square test with Bonferroni correction, except when the expected frequency in one or more cells was less than 5, in which case Fisher's exact test was applied.

* A p-value of P < 0.05 is considered significant, with directionality b is significantly higher than a.

### 3.2 Agreed-events and interrater agreement

In the *AVG* montage detection, 9555 (69.6 %) agreed-IEDs, 1791 (68.9 %) agreed-ripples, and 42 (57.1 %) agreed-FRs were retained (see Table 2 for recording and patient numbers). In the *HBIP* montage detection, 9003 (66.0 %) agreed-IEDs, 2098 (63.8 %) agreed-ripples, and 44 (60.7 %) agreed-FRs were retained. In the *VBIP* montage detection, 7624 (68.6 %) agreed-IEDs, 1814 (55.2 %) agreed-ripples, and 45 (53.0 %) agreed-FRs were retained.

The overall weighted average κ-value for the *AVG* montage was 0.70 (IEDs: 0.69, ripples: 0.71, FRs: 0.75); for the *HBIP* 0.66 (IEDs: 0.66, ripples: 0.67, FRs: 0.71); and for the *VBIP* 0.68 (IEDs: 0.69, ripples: 0.60, FRs: 0.65). All κ-values exceeded the predefined threshold of 0.5, indicating reliable interrater agreement between the two reviewers (ZW and JG). After Tukey’s adjustment, the GLMM pairwise comparisons showed that the two reviewers achieved a higher κ-value on ripple-marking in *AVG* and *HBIP* compared to the *VBIP* montage ((*AVG vs. VBIP Estimate (ES)* = 0.19, *p_Tukey_* < 0.0001; *HBIP vs. VBIP ES* = 0.12, *p_Tukey_* = 0.01)). There was no difference in κ-value for IEDs- and FRs-marking among the three montages (all *p_Tukey_* > 0.05).

More channels with agreed-ripples were observed in *BIP* montages (both *H&V*) compared to *AVG* montage (*HBIP vs. AVG p_bonf_ <* 0.0001, *VBIP vs. AVG p_bonf_ <* 0.0001). No difference in the number of channels with agreed-IEDs and −FRs was found. There were no differences among the three montages in the number of patients with agreed-events (IEDs & HFOs), nor in the number of recordings with agreed-events (Table 2).

### 3.3 Montage performance

#### 3.3.1 Number of channels-with-events

**Fig. 2:**
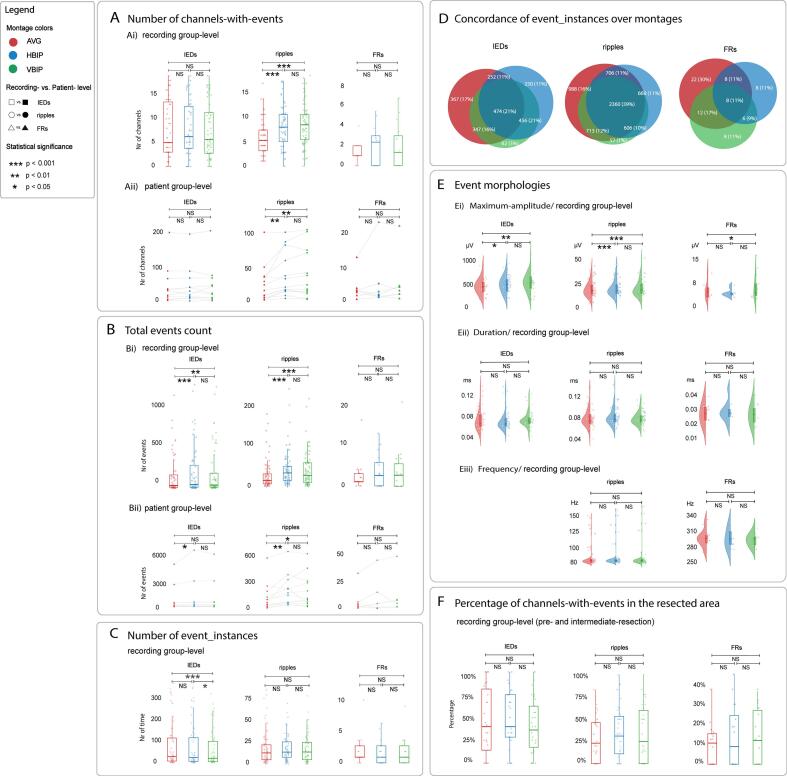
**Comparison of montage performance across *AVG*, *HBIP*, and *VBIP* montages. A. Number of channels-with-events. Ai)** at recording- and **Aii)** patient- group level, *BIP* montages (both *H&V*) yielded significantly more channels with ripples than an *AVG* montage. (recording group-level: *HBIP vs. AVG p* < 0.0001, *VBIP vs. AVG p* < 0.0001; patient group-level: *HBIP vs. AVG p* = 0.003, *VBIP vs. AVG p* = 0.005). **B. Total events count. Bi)** at recording group-level, *BIP* montages (both *H&V*) yielded significantly more IEDs and ripples compared to an *AVG* montage (*HBIP vs. AVG* IEDs *p* = 0.0001, ripples *p* < 0.0001; *VBIP vs. AVG*, IEDs *p* = 0.008, ripples *p* < 0.0001); **Bii)** at patient group-level, *BIP* montages (both *H&V*) yielded more significantly ripples compared to an *AVG* montage (*HBIP vs. AVG p* = 0.002, *VBIP vs. AVG p* = 0.02)**;** an *HBIP* montage yielded significantly more IEDs compared to an *AVG* montage (*p* = 0.02). **C. Number of event_instances.** At recording-group level, both *AVG* and *HBIP* montages yielded significantly more IED_instances compared to a *VBIP* montage (*AVG vs. VBIP p* = 0.0002; *HBIP vs. VBIP p* = 0.05). **D. Concordance of event_instances over montages.** Scaled Venn plots per event type displaying the number (% of total event_instances) of concordant event_instances at each inter- and divergence section across the three montages. An *AVG* montage yielded the highest percentage of unique event_instances: 17 % IEDs, 16 % ripples, and 30 % FRs. A *VBIP* montage yielded the lowest percentage of unique event_instances with 3 % IEDs, and 1 % ripples. **E. Event morphology. Ei)** at recording group-level, *BIP* montages (both *H&V*) yielded a significantly higher maximum-amplitude for IEDs and ripples than those in an *AVG* montage (*HBIP vs. AVG* IEDs *p* = 0.02, ripples *p* = 0.0001; *VBIP vs. AVG* IEDs *p* = 0.0003, ripples *p* = 0.003); A *VBIP* montage yielded a significantly higher maximum-amplitude for FRs in comparison to those in an *AVG* montage (*p* = 0.002). **Eii)** duration and **Eiii)** frequency analysis showed no significant differences for IEDs and HFOs among the three montages at recording group-level. **F. Percentage of channels in pre- and intermediate-resection recordings with events located within the resected-area**: There were no significant differences in IEDs and HFOs among the three montages. Statistical significances at the patient group-levels (**Aii)** and **Bii)**) were determined using the Friedman test, with the Nemenyi test applied as a post-hoc analysis. Statistical significances at the recording group-levels were determined by a GLMM (model 1 for panel **E**; model 2 for panel **Ai)**, **Bi)**, **C**, **F**) with Tukey adjustment. The inner horizontal line marks the median, and the inner box indicates the standard deviation (25th to 75th percentiles). *Abbreviations*: *AVG* = *average* montage; *HBIP* = horizontally-oriented *bipolar* montage; *VBIP* = vertically-oriented *bipolar* montage; IED = interictal epileptiform discharge; FR = fast ripple; GLMM = generalized linear mixed model; HFOs = high frequency oscillations; NS = not significant.

After channel normalization, *BIP* montages *(*both *H&V)* yielded more channels-with-ripples compared to *AVG* montage at recording- and patient- group levels (GLMM **|** *HBIP vs. AVG ES* = 0.33, *p_Tukey_* < 0.0001, *VBIP vs. AVG ES* = 0.37, *p_Tukey_* < 0.0001; Friedman **|** *HBIP vs. AVG p_Nemenyi_* = 0.003, *VBIP vs. AVG p*_Nemenyi_ = 0.005; Fig. 2A). We found no difference in the count of channels-with-ripples between the *HBIP* and *VBIP* montages (Fig. 2A). We found no differences in the numbers of channels-with-IEDs or −FRs among the three montages.

#### 3.3.2 Total event counts

*BIP* montages (both *H&V*) yielded more IEDs and ripples compared to *AVG* montage at the recording group-level (GLMM **|** *HBIP vs. AVG* IEDs *ES* = 0.23, *p_Tukey_* = 0.0001, ripples *ES* = 0.45, *p_Tukey_* < 0.0001; *VBIP vs. AVG* IEDs *ES* = 0.17, *p_Tukey_* = 0.008, ripples *ES* = 0.40, *p_Tukey_* < 0.0001; Fig. 2Bi)). At the patient group-level, *HBIP* montage yielded more IEDs compared to *AVG* montage (Friedman **|** *p_Nemenyi_* = 0.02); both *BIP* montages (*H&V*) yielded more ripples compared to an *AVG* montage (Friedman **|** *HBIP vs. AVG p_Nemenyi_* = 0.002, *VBIP vs. AVG p_Nemenyi_* = 0.02; Fig. 2Bii). We found no differences in IED and ripple counts between the *HBIP* and *VBIP* montages (Fig. 2B) and in FR counts among the three montages.

#### 3.3.3 Number of event_instances

Both *AVG* and *HBIP* montages yielded more IED_instances compared to a *VBIP* montage (GLMM **|** *AVG vs. VBIP ES* = 0.24, *p_Tukey_* = 0.0002; *HBIP vs. VBIP ES* = 0.14, *p_Tukey_* = 0.05; Fig. 2C). We found no difference in the number of IED_instances between *HBIP* and *AVG* montages and in the number of ripple_or FR_instances among the three montages.

#### 3.3.4 Concordance of event_instances over montages

The percentage of “concordant event_instances” that occurred simultaneously across all three montages was 21 % for IED, 39 % for ripple, and 11 % for FR.

The percentage of “unique event_instances” in *AVG* montage was 17 % for IEDs, 16 % for ripples, and 30 % FRs, in *HBIP* montage 11 % of IED_, ripple_, and FR_instances; and in *VBIP* 3 % of IED_, 1 % of ripple_, and 11 % of FR_instances (Fig. 2D).

#### 3.3.5 Event morphology

Fig. 2E shows the differences in event morphology across the three montages at the recording group-level. The amplitudes of IEDs and ripples were higher in both *HBIP* and *VBIP* montages compared to *AVG* montage. (GLMM **|** *HBIP vs. AVG* IEDs *ES* = 0.09, *p_Tukey_* = 0.02, ripples *ES* = 0.11, *p_Tukey_* = 0.0001; *VBIP vs. AVG* IEDs *ES* = 0.13, *p_Tukey_* = 0.0003, ripples *ES* = 0.09, *p_Tukey_* = 0.003); A *VBIP* montage yielded higher FRs amplitudes than an *AVG* montage (GLMM **|** *ES* = 0.17, *p_Tukey_* = 0.002). There were no differences in the amplitudes of IEDs, ripples, or FRs between *HBIP* and *VBIP* montages.

No differences in durations and frequency were observed among the three montages for IEDs, ripples, and FRs.

#### 3.3.6 Percentage of channels-with-events located within the resected-area

We found no differences in the percentage of channels-with-events (IEDs, ripples, and FRs) covering the resected tissue during pre- and intermediate-resection recordings among the three montages (Fig. 2F).

## 4 Discussion

We investigated the optimal montage in marking ioECoG biomarkers (i.e., IEDs and HFOs) and came up with three main findings: 1) *Bipolar* montages, regardless of electrode connection direction, yielded more channels, higher counts, and greater amplitudes of ripples and IEDs compared to *average* montages. 2) *Average* montages showed most unique IEDs_and HFOs_instances which were not identified in one of the *bipolar* montages. 3) The percentage of IEDs and HFOs channels covering the epileptogenic tissue was similar in *average* and *bipolar* montages.

During ioECoG tailored epilepsy surgery, prompt decision-making is required. An *average* montage may provide a faster and straightforward overview, whereas *bipolar* montages may require more time for detailed analysis. We observed that an *average* montage yielded greater raw counts of IEDs and ripples than an *bipolar* montage (Table 2). Given the configuration by us used 4x5 electrode grids, an *average* montage comprised more channels (20 channels) than an *bipolar* montage (16 channels in horizontal-orientation and 15 in vertical). This emphasizes the importance of conducting comparisons on an equal basis, accounting for differences in the number of channels between montages. After channel normalization, the results reversed: higher counts of IED and ripple channels, and greater event numbers were found in the *bipolar* montages. A possible explanation is that an electrode pair in a *bipolar* montage records both the input and output sides, (Acharya et al., 2016, Zaveri et al., 2006) resulting in the same event being marked twice. Two duplicate events, observed as phase reversals in adjacent channels of a bipolar montage, are often optimal for marking due to their distinct localization and prominence. (Acharya and Acharya, 2019, Pickard and Skidmore, 2019).

We found that *bipolar* montages displayed more distinct amplitudes of IEDs and ripples, which may facilitate visual discrimination, compared to the *average* montage. This is probably because the *bipolar* montage emphasized local voltage differences between two adjacent electrodes, reducing the influence of widespread distant noise. (Pickard and Skidmore, 2019) In contrast, an *average* montage computes the mean voltage across all channels. During this procedure channels contaminated with artefacts need to be removed; (Acharya and Acharya, 2019) The averaging method may dilute extensive IEDs or oscillations, often resulting in lower amplitude signals that might cause them to fail to meet the strict criteria for event marking. The other way around, focal high amplitude events can be reflected on other channels. Above reasons may make the *bipolar* montage superior to *average* montage for subtle event analysis where clean, localized signals are crucial.

In our center, we are used to review the ioECoG using a horizontal-orientation *bipolar* montage for research purposes, (Schaft et al., 2024, Sun et al., 2024, van't Klooster et al., 2015, van't Klooster et al., 2017, van Klink et al., 2014, van Klink et al., 2021) but when needed we create an *average* montage to confirm whether the observed events are truly present and to rule out distortions from *bipolar* subtraction. For clinical evaluation of IEDs during the surgical procedure, we most often use the *average* montage. In this study, we found an *average* montage added some new information to the horizontally-oriented *bipolar* montage (17 % unique IED_instances, 16 % ripple_instances, 30 % FR_instances), while the vertically-oriented *bipolar* montage added less (3 % unique IEDs_instances, 1 % ripple_instances, 11 % FR_instances). One interpretation might be that some events are canceled out in a *bipolar* montage if they occur relatively synchronously at the two paired electrodes which may be picked up by the *average* montage. Another interpretation is that if an electrode in the middle of the grid is noisy, one channel will be excluded from further analysis in an *average* montage, whereas two channels will be excluded in a *bipolar* montage. Moreover, electrodes positioned in the middle of the grid benefit from having neighboring electrodes on all sides, enabling multiple bipolar pairs in two or more traces for each electrode. In contrast, the edges and corners electrodes lack this advantage, making it difficult to discern whether the electrical activity originates from one electrode or is equally shared between both. The vertically-oriented configuration can preserve events on the lateral and corner channels when the horizontally-oriented channels are contaminated with artifacts and vice versa. This is illustrated in Fig. 1Biii; as Gr17 is contaminated by high-frequency noise, events displayed by Gr16 are also missed in the horizontally-oriented *bipolar* configuration; whereas the Gr11-Gr16 channel in vertically-oriented *bipolar* montage still captures these events.

Brain activity can propagate differently across sulci due to differences in cortical connectivity. Placing electrodes on the same gyrus allows for a clearer understanding of localized IEDs and HFOs because neighboring electrodes are sampling the same cortical area. Nonetheless, in 15 out of 79 recordings, particularly those involving the temporal lobe, the grids were often aligned with the direction of gyri. This alignment resulted in pairs of channels from the vertically-oriented *bipolar* montage to go across sulci and detect events from different gyri. A region of HFOs rather covers a single gyrus than multiple adjacent gyri; This could be reflected by multiple consequential channels showing HFOs in horizontal direction and just single channels in vertical direction. The ambiguity about the precise origin of the vertically detected ripple may have made it more challenging for both reviewers (ZW, JG) to confidently mark the events. This likely contributed to lower agreement on whether the ripple was truly present and distinct (κ-value 0.60 for ripples in *VBIP*). Consequently, fewer unique event_instances (3 % IEDs, 1 % ripples) were detected in the vertical configuration.

Calculating the percentage of channels with IEDs or HFOs inside the assumed epileptogenic zone is a widely adopted approach to evaluate their predictive value in epilepsy surgery research (Kural et al., 2020b, Shi et al., 2024, van't Klooster et al., 2017). Complete resection of areas generating IEDs and the absence of residual IEDs after resection has been associated with favorable seizure outcome (Ferrier et al., 2006, Guo et al., 2024). A recent meta-analysis showed that fully HFOs removal, especially that of FRs, correlates with good seizure outcome (Wang et al., 2024). Would different montages change the distribution of channels-with-events and in turn affect the diagnostic value for locating epileptogenic tissue? This study answered that question by showing no significant differences between the *average* and *bipolar* montages in the percentage of channels with IEDs and HFOs covering the epileptogenic tissue. In other words, the distribution of channels showing IEDs and HFOs in the epileptogenic tissue was robust and unaffected by the choice of the ioECoG montage.

A strength of our study is its contribution to bridge practical research gap in investigating which ioECoG montage is optimal for marking IEDs and HFOs. We provided quantitative data to evaluate these differences addressing a broad spectrum of aspects for its use in clinical practice (e.g. number of channels-with-events, total events count, event morphology, concordance of event_instances over montages, and percentage of channels-with-events in the resected-area). Another strength is that the IEDs and HFOs analyzed in this study were co-labeled by two observers. The acceptable κ-value (all > 0.60) confirms the reliability of these events. Finally, we evaluated an alternative vertically-oriented *bipolar* montage and demonstrated that it added less unique information to other two montage.

The study’s limitations include the small sample size for FRs identification and the definition of epileptogenic tissue. The rarity of FRs and the low sensitivity of standard equipment resulted in their small size, (van't Klooster et al., 2017) necessitating optimized marking strategies by multiple montages. Determining the exact boundaries of the epileptogenic zone is difficult even if postoperative seizure freedom was achieved. It is possible that some channels within the assumed epileptogenic tissue were covering non-epileptic, but resected tissue. The number of patients was low to rule out a small difference in the value to localize the epileptic tissue. As our initial goal was to find an optimal montage for identifying specific events, we deliberately did not compare the localization accuracy of IEDs to that of HFOs. In addition, we did not take the event-threshold into account: regardless of whether a channel had one event or a dozen, it was classified as a “channel-with-events” in present work. In clinical practice, channels with only one or very few abnormalities might be less relevant identifier of the seizure focus than channels with frequent events. Future studies should focus on setting a specific threshold to determine whether a channel should be considered for resection, rather than simply labeling it as 'abnormal' based on the presence of IEDs or HFOs.

## 5 Conclusion and future recommendations

All three ioECoG montages holds value in clinical utility to find epileptic events. The *bipolar* montage excels at detecting a higher number of events with greater amplitude, and the *average* reveals a broader spectrum of unique events. Rather than favoring one montage over the others, the choice to review the ioECoG between *bipolar* or *average* montage should align with specific goals, data nature, and personal preferences. Notably, combining these montages offers complementary insights. Future advancements in automated systems could integrate data from both *bipolar* and *average* montages, enabling comprehensive analyses across multiple approaches. This progression would unify information, reduce the effort for manual review, and enhance localization accuracy. Furthermore, it has been suggested that high-density recordings could enhance the signal-to-noise ratio and increase the possibility of HFO detections. Further research is needed to investigate whether current results for standard grids can be generalized to high-density grids.

## 6 Disclosure

The authors report no disclosures relevant to the manuscript.

## Declaration of competing interest

The authors declare that they have no known competing financial interests or personal relationships that could have appeared to influence the work reported in this paper.
